# Erythrodermic psoriasis with bullous pemphigoid: combination treatment with methotrexate and compound glycyrrhizin

**DOI:** 10.1186/1746-1596-9-102

**Published:** 2014-05-29

**Authors:** Xiaoqing Si, Lingzhi Ge, Hongyan Xin, Wang Cao, Xiaohui Sun, Wenfei Li

**Affiliations:** 1 Department of Dermatology, Qianfoshan Hospital, Shandong University, Jinan 250014, China; 2 Taishan Medical College, Tai’an 271000, China; 3 Shandong Chest Hospital, Jinan 250013, China; 4 Department of Dermatology, Jinan Sixth People’s Hospital, Jinan 250200, China

**Keywords:** Erythrodermic psoriasis, Bullous pemphigoid, Methotrexate, Compound glycyrrhizin

## Abstract

We report a case of erythrodermic psoriasis with bullous pemphigoid (BP) in a 68-year-old male. The patient had a history of psoriasis for 35 years and tense, blisterlike lesions for 4 months. He presented with diffuse flushing, infiltrative swelling, and tense blisterlike lesions on his head, trunk, and limbs. This patient was successfully treated by a combination of methotrexate and compound glycyrrhizin. We also discuss the clinical manifestations, histopathological features, and differentiation of erythrodermic psoriasis with BP and present a review of the pertinent literature.

**Virtual Slides:**

The virtual slide(s) for this article can be found here: http://www.diagnosticpathology.diagnomx.eu/vs/1853737109114076

## Background

Erythrodermic psoriasis is an uncommon, severe form of psoriasis, accounting for about 1% of psoriatic patients. BP predominantly affects older people, with clinical features of tense bullae on trunk and limbs. By immunoblot analysis, 200-kDa or 180-kDa antigen identified in dermal extracts have been shown to play a major role in patients with psoriasis and BP [1]. Anti-laminin-γ1 pemphigoid (ALγ1P), a new kind of autoimmune BP, was detected as a 200-kDa pathogenic antigen in dermal extracts. Half the ALγ1P cases were reported to be associated with psoriasis [2]. Coexistence of erythrodermic psoriasis with BP is rare. To the best of our knowledge, only two cases of such association have been described.

Here we report a case of a 68-year-old male who suffered from erythrodermic psoriasis with BP who was successfully treated with a combination of methotrexate and compound glycyrrhizin.

## Case presentation

**Figure 1:**
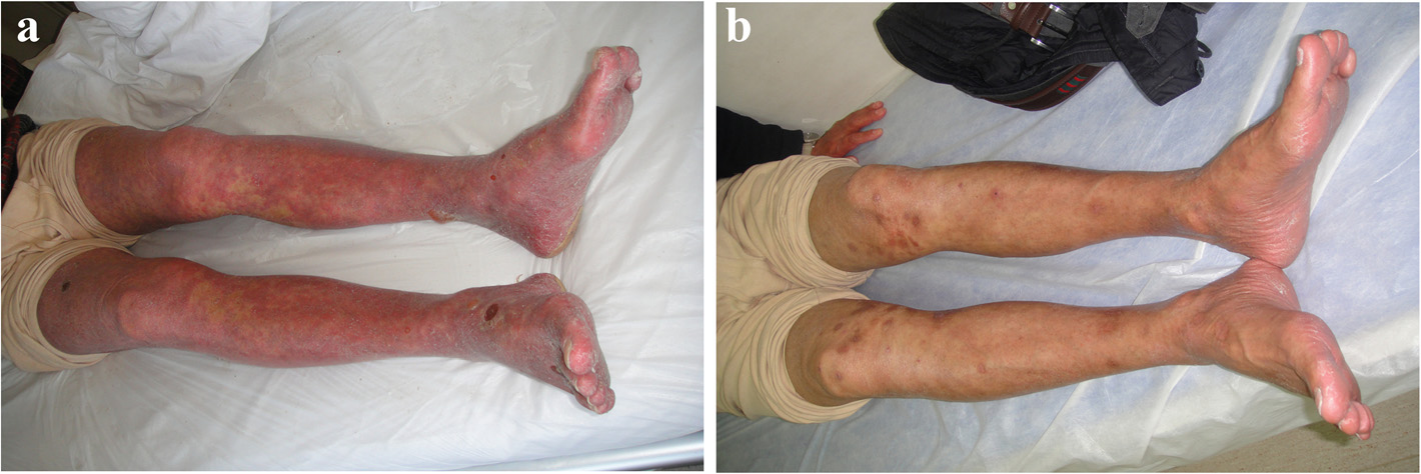
**Topical rash before treatment and normal condition after treatment on his limbs and feet. (a)** Diffuse flushing, tense, transparent vesicles, and blisters on his limbs and feet. Skin lesions showing diffuse flushing, infiltrative swelling, a large number of branlike scales on his limbs, and tense, transparent vesicles and blisters on his feet. **(b)** Diffuse flushing and infiltrative swelling has subsided. Skin lesions cleared and normal condition restored.

A 68-year-old Chinese male complained of a history of psoriasis for more than 30 years and tense, blisterlike lesions for 4 months. He had been prescribed multiple therapeutic measures, and after he was treated with narrow-spectrum ultraviolet on October 17, 2012, the tense, blisterlike lesions were noticed on the distal limbs, particularly the feet. The patient was treated with prednisone 30 mg daily, and his condition was gradually brought under control, but suddenly worsened after a week of discontinuing prednisone. He is an engineer who has had no history of smoking, drinking, hypertension, or diabetes. Physical examination showed diffuse flushing, infiltrative swelling, tense transparent vesicles and blisters on his head, trunk, and limbs. There was palmoplantar desquamation with glove- and sock-like distribution. After peeling, the skin was meager and bright red (Figure 1a). Bulla spread and Nikolsky’s signs were negative. There was no evidence of any cardiac, pulmonary, hepatic, nephritic, or central nervous system involvement evidenced by electrocardiogram, chest X-ray, abdominal ultrasound examination, or cranial computed tomography.

**Figure 2:**
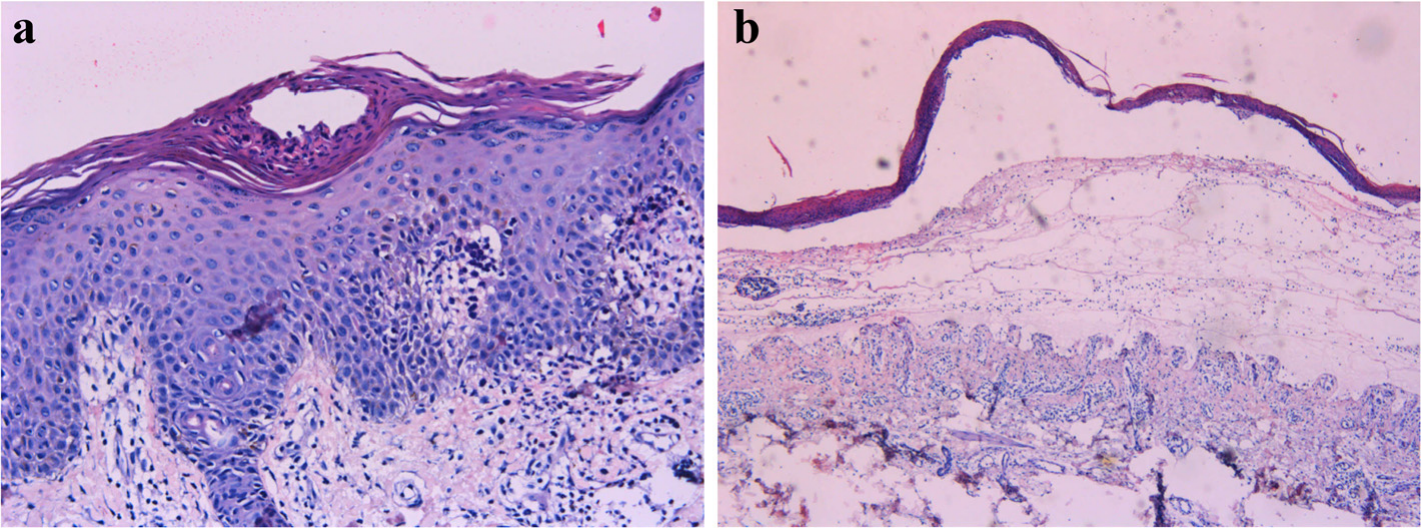
**Characteristics of skin histopathology supporting the diagnoses of psoriasis and BP. (a)** Munro’s microabscesses, hypogranulosis, and dilated blood vessels in the superficial dermis. Skin biopsy showing hyperkeratosis, parakeratosis, Munro’s microabscesses, hypogranulosis, acanthosis with regular elongation of rete pegs, and congested and dilated blood vessels in the superficial dermis (H&E, ×200). **(b)** Blister located subepidermally. Subepidermal blister filled with serous exudate, with eosinophil-rich inflammatory infiltrates at the bottom of the blister (H&E, ×100).

**Figure 3:**
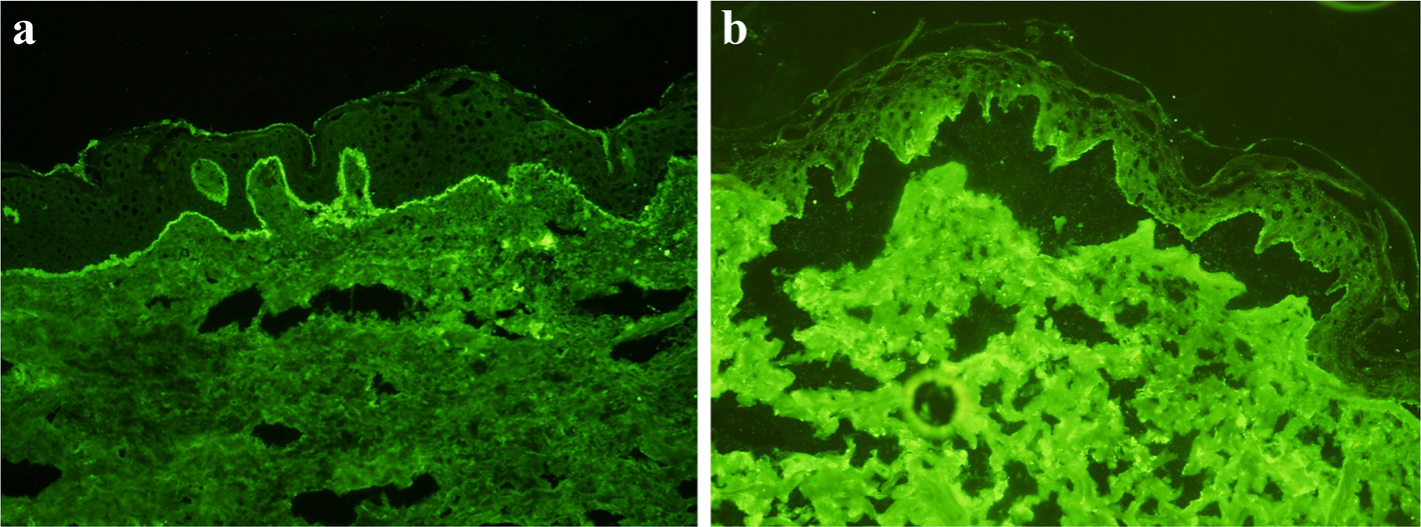
**Immunofluorescence revealing histopathologic characteristics of bullous pemphigoid. (a)** IgG and C3 deposited along the basement membrane. Direct immunofluorescence revealing IgG and C3 deposited along the basement membrane (×100). **(b)** IgG deposited on the epidermal side. Indirect immunofluorescence of normal human skin showing IgG deposited on the epidermal side (×200).

Laboratory analysis revealed a white blood cell count of 6.12 × 10^9^/L (normal: 3.97–9.15 × 10^9^/L) with 1.19 × 10^9^/L eosinophils (normal: 0.00–0.50 × 10^9^/L); hemoglobin 122.0g/L (131–172 g/L). There was an elevated erythrocyte sedimentation rate (ESR) of 18 mm/h (normal: 0–15 mm/h); C-reactive protein (CRP) level was 28.1 mg/L (normal 0–3 mg/L); circulating antibodies to BP180 NC16A and BP230-gC were positive in the peripheral blood serum.Histopathological biopsy taken from the left forearm showed hyperkeratosis, parakeratosis, Munro’s microabscess, hypogranulosis, and acanthosis with regular rete elongation, and congested and dilated blood vessels in the superficial dermis (Figure 2a). There was subepidermal blistering with eosinophil-rich inflammatory infiltrates at the bottom of the blister, which was typical for BP (Figure 2b). Direct immunofluorescence revealed immunoglobulin G (IgG) and C3 deposited along the basement membrane (Figure 3a). Indirect immunofluorescence on normal human skin showed that IgG was deposited on the epidermal side (Figure 3b). Based on clinical and histopathological features, a final diagnosis of erythrodermic psoriasis with BP was made.The patient was treated with methotrexate at a dose of 15 mg weekly and compound glycyrrhizin 150 mg daily. After 2 weeks, the patient’s condition had improved, and the diffuse flushing and the infiltrative swelling had subsided (Figure 1b). He was in clinical remission at 6 months’ follow-up. All hematological and biochemical examinations were normal and no new lesions were noticed.

## Discussion

The pathogenesis of erythrodermic psoriasis with BP is not fully understood, but much investigation has been done on the pathogenetic association between psoriasis and BP when treated with sartan drugs, including losartan-induced BP in a psoriatic patient [3]. After receiving PUVA or UVB therapy for psoriasis, many patients presented with bullous lesions, so ultraviolet therapy was regarded as a possible precipitating factor in triggering bullous lesions in psoriatic patients. The mechanism was thought to be that PUVA or UVB raised the immunogenecity of the basement membrane proteins, leading to a higher risk of the autoantibody formation [4].

Certain clinical features are used to differentiate psoriasis with BP from other diseases. Seborrheic dermatitis often affects the scalp, face, and torso. Typically, it affects the sebaceous gland–rich areas of the skin. In adolescents and adults, it usually presents as scalp scaling similar to dandruff or as mild to marked erythema of the nasolabial fold [5]. Sometimes psoriatic lesions are similar to those of chronic eczema. However, there are some telltale signs: psoriasis tends to involve the elbows and knees, but eczema favors the inside of the arms and back of the knees. BP and epidermolysis bullosa acquisita have many common characteristics, such as proclivity for the elderly and presence of tense and subepidermal blisters. But in our patient, indirect immunofluorescence of BP on normal human skin showed IgG deposited on the epidermal side. Circulating antibodies to BP180 NC16A and BP230-gC were positive in the serum of peripheral blood [6]. By these features, BP can be differentiated from epidermolysis bullosa acquisita.

Erythrodermic psoriasis and BP seldom coexist in the same patient. To the best of our knowledge, only two cases of such association have been described. One patient received cyclosporine therapy combined with systemic steroids [7]. Another was successfully treated with a combination of acitretin and azathioprine [8]. We treated our patient with a combination of methotrexate and compound glycyrrhizin.

Methotrexate has been shown to have a significant therapeutic effect in the treatment of psoriasis [9]. A recent retrospective review of 710 outpatients who used methotrexate to treat moderate to severe psoriasis demonstrated that methotrexate is relatively safe [10]. In the past 10 years, compound glycyrrhizin used in the clinic reduced the activity of the T-lymphocyte subset, helped recover lymphocytes, and had satisfactory efficacy and high safety in the treatment of psoriasis vulgaris. Whenever we applied compound glycyrrhizin to treat a patient suffering from recurrent cutaneous necrotizing eosinophilic vasculitis, we got a good result [11]. With a combination of methotrexate and compound glycyrrhizin, we can quickly clear both erythrodermic psoriasis and BP lesions. Some studies have found that interleukin 8 (IL-8) plays an important role not only in inflammatory acne vulgaris [12], but also in psoriasis and bullous pemphigoid [13,14], so compound glycyrrhizin might block production of some cytokines, including IL-8, in the treatment of erythrodermic psoriasis and bullous pemphigoid lesions. We received valuable experience from the first case using this combined treatment, which encouraged us to continue these clinical trials for such patients.

## Conclusion

Erythrodermic psoriasis with BP has a low incidence of coexisting in the same patient. We have shown that methotrexate and compound glycyrrhizin can be an effective alternative therapy in the treatment of erythrodermic psoriasis with BP, but long-term prospective studies of this regimen and an adequate sample size are required to assure its safety and efficacy.

## Consent

Written informed consent was obtained from the patient for publication of this case report and all accompanying images. A copy of the written consent is available for review by the editor-in-chief of this journal.

## Abbreviations

BP: Bullous pemphigoid; ALγ1P: Antilaminin-γ1 pemphigoid; PUVA: Psoralen with ultraviolet A; UVB: Ultraviolet B; ESR: Erythrocyte sedimentation rate; CRP: C-reactive protein; Ig G: Immunoglobulin G; IL-8: Interleukin 8.

## Competing interest

The authors declare that they have no competing interests.

## Authors’ contributions

WFL designed the study, performed the histological evaluation, and wrote the paper; XQS and LZG were involved in the literature search and preparing the material; HYX and WC participated in the histological diagnosis; XHS participated in providing the clinical information of the case. All authors read and approved the final manuscript.

## References

[B1] Yasuda H Tomita Y Shibaki A Hashimoto T Two cases of subepidermal blistering disease with anti-p200 or 180-kD bullous pemphigoid antigen associated with psoriasis Dermatology 2004 209 149 155 10.1159/000079602 15316172

[B2] Majima Y Yagi H Tateishi C Groth S Schmidt E Zillikens D Koga H Hashimoto T Tokura Y A successful treatment with ustekinumab in a case of antilaminin-γ1 pemphigoid associated with psoriasis Br J Dermatol 2013 168 1367 1369 10.1111/bjd.12163 23252972

[B3] Saraceno R Citarella L Spallone G Chimenti S A biological approach in a patient with psoriasis and bullous pemphigoid associated with losartan therapy Clin Exp Dermatol 2008 33 154 155 10.1111/j.1365-2230.2007.02603.x 18021271

[B4] Kirtschig G Chow ET Venning VA Wojnarowska FT Acquired subepidermal bullous diseases associated with psoriasis: a clinical, immunopathological and immunogenetic study Br J Dermatol 1996 135 738 745 10.1111/j.1365-2133.1996.tb03883.x 8977674

[B5] Kim GW Jung HJ Ko HC Kim MB Lee WJ Lee SJ Kim DW Kim BS Dermoscopy can be useful in differentiating scalp psoriasis from seborrhoeic dermatitis Br J Dermatol 2011 164 652 656 21155753 10.1111/j.1365-2133.2010.10180.x

[B6] Schmidt E Zillikens D Modern diagnosis of autoimmune blistering skin diseases Autoimmun Rev 2010 10 84 89 10.1016/j.autrev.2010.08.007 20713186

[B7] Bianchi L Gatti S Nini G Bullous pemphigoid and severe erythrodermic psoriasis: combined low-dose treatment with cyclosporine and systemic steroids J Am Acad Dermatol 1992 27 2 Pt 1 278 1430380 10.1016/s0190-9622(08)80749-9

[B8] Roeder C Driesch PV Psoriatic erythroderma and bullous pemphigoid treated successfully with acitretin and azathioprine Eur J Dermatol 1999 9 537 539 Review 10523731

[B9] Mahajan VK Sharma AL Chauhan PS Mehta KS Sharma NL Early treatment with addition of low dose prednisolone to methotrexate improves therapeutic outcome in severe psoriatic arthritis Indian J Dermatol 2013 58 240 23723489 10.4103/0019-5154.110847 PMC3667301

[B10] Ng LC Lee YY Lee CK Wong SM A retrospective review of methotrexate-induced hepatotoxicity among patients with psoriasis in a tertiary dermatology center in Malaysia Int J Dermatol 2013 52 102 105 10.1111/j.1365-4632.2011.05436.x 23278617

[B11] Li W Cao W Song H Ciu Y Lu X Zhang F Recurrent cutaneous necrotizing eosinophilic vasculitis: a case report and review of the literature Diagn Pathol 2013 8 185 10.1186/1746-1596-8-185 24199910 PMC3874652

[B12] Abd El All HS Shoukry NS El Maged RA Ayada MM Immunohistochemical expression of interleukin 8 in skin biopsies from patients with inflammatory acne vulgaris Diagn Pathol 2007 2 4 10.1186/1746-1596-2-4 17263887 PMC1797156

[B13] Kim HO Kim JH Chung BY Choi MG Park CW Increased expression of the aryl hydrocarbon receptor in patients with chronic inflammatory skin diseases Exp Dermatol 2014 23 278 281 10.1111/exd.12350 24521260

[B14] Van den Bergh F Eliason SL Burmeister BT Giudice GJ Collagen XVII (BP180) modulates keratinocyte expression of the proinflammatory chemokine, IL-8 Exp Dermatol 2012 21 605 611 10.1111/j.1600-0625.2012.01529.x 22775995 PMC3395233

